# PROF. PHD. HENRIQUE WALTER PINOTTI: 1929 - 2010. FOUNDER OF THE BRAZILIAN COLLEGE OF DIGESTIVE SURGERY

**DOI:** 10.1590/0102-672020210002e1669

**Published:** 2022-07-29

**Authors:** Ivan CECCONELLO, Marco Aurélio SANTO

**Affiliations:** 1 Professor of Surgery, Medical School of the University of São Paulo - USP, Brazil. Full Member and former President of the Brazilian College of Digestive Surgery (CBCD).; 2 Professor of Surgery, Medical School of the University of São Paulo - USP, Brazil. Full Member of the Brazilian College of Digestive Surgery (CBCD)

Henrique Walter Pinotti was born in São Paulo to Italian immigrants. He began attending the Hospital das Clínicas of the Medical School of the University of São Paulo (FMUSP) when he was admitted to the Medical School, in 1950, participating in the Clinical Gastroenterology Service as an academic trainee under the guidance of the then head, Professor José Fernandes Pontes. He went through all sectors of the specialty, having contact with the pathophysiological and clinical bases of gastroenterology, deciding on surgery on the eve of his graduation, in December 1955. Then, he was selected as a second-year resident physician of the Department of Surgery of the Hospital, a position held until May 1958. During those years, he also attended the Department of Pathological Anatomy as a volunteer.

He was granted his first academic title in March 1959, when he was appointed by Professor Alípio Corrêa Netto, upon recommendation of Professor Arrigo Raia, Teaching Assistant of Surgical Clinics. Later, in 1960, he was appointed FMUSP Assistant.

Since then, he had the opportunity to monitor the activities and absorb the teachings of renowned surgeons, notable school leaders, such as Benedito Montenegro, Edmundo Vasconcellos, Eurico da Silva Bastos and, especially, Alípio Corrêa Netto, from whom he received great encouragement for his university career, as well as from his followers, Euryclides de Jesus Zerbini and Arrigo Raia, with whom he enjoyed a close and long-term relationship.

He presented his Doctoral Dissertation at FMUSP in 1964 and became an Associate Professor, by public official examination, in Surgical Clinic in 1967, and the dissertations of both programs were related to Megaesophagus.

In 1968, a new academic order was established: the chairs were extinguished and Departments were created. Disciplines and departments were established; the former, in fact, are specific teaching programs, and the later, the bunion of related disciplines. At that moment, the Discipline of Digestive Surgery was established at FMUSP, as well as the respective Digestive Surgery Division at the Hospital das Clínicas, to house surgeons specialized in this area from the former chairs.

He always sought to follow the specialty in its entirety, performing activities in all areas. With the structuring of this Discipline, he assumed, from 1974 onwards, the Head of the Esophageal Surgery Service, where he promoted great development to Brazilian esophagology.

In 1981, he obtained the title of Adjunct Professor of FMUSP, and in August 1982, with the retirement of Professor Arrigo Raia, upon recommendation of the Board of the Department of Surgery, he held the position of Professor of the Discipline of Digestive Surgery of FMUSP. Subsequently, on recommendation of the same Board, he was appointed Technical Director of the Digestive Surgery Division of Hospital das Clínicas and its services: surgeries of esophagus, stomach, and small intestine, liver and portal hypertension, colon, rectum, and anus. In 1984, after a memorable civil service competitive examination, he was appointed Full Professor of Digestive Surgery at FMUSP.

A prominent event took place in 1986, with the establishment of the Department of Gastroenterology of the FMUSP, on the initiative of Professors Agostinho Bettarello and Henrique Walter Pinotti. This event was important for the national and international history of clinical and surgical Gastroenterology and also for teaching students, residents, and graduate students, as well as for conducting scientific and care-related research with the integration of surgeons, gastroenterologists, endoscopists, and nutrologists.

In his memorial, the following stand out: 590 publications in national and international journals; 26 awards; 46 films or videos and countless lectures at national and international congresses and courses; 191 book chapters and seven books, among which stand out *Tratado de Cirurgia do Aparelho Digestivo*, *Acesso ao Esôfago Torácico por Transsecção Mediana do Diafragma*, and *Filosofia da Cirurgia* (Textbook of Surgery of the Digestive System, Access to the Thoracic Esophagus by Median Diaphragm Transection, and Philosophy of Surgery)^2^ ^,^ ^3^ ^,^ ^4^ ^,^ ^5^ ^,^ ^6^ ^,^ ^7^ ^,^ ^8^ ^,^ ^9^.

In 1973, he was founder, together with Professors Arrigo Raia, Joaquim José Gama-Rodrigues, Marcel Cerqueira Cesar Machado, and Angelita Habr-Gama, of the Digestive Surgery Update Course (*Curso de Atualização de Cirurgia do Aparelho Digestivo - Gastrão*), the most important in Digestive Surgery in Brazil. He has always been intensely dedicated to this course, especially during his tenure. It is also noteworthy, in 1974, the creation of the Continuing Course in Gastroenterology (*Curso Continuado em Gastroenterologia*), periodically administered in 10 annual sessions, on Saturdays, for doctors from the State of São Paulo and Brazil.

In 1986, he created the Brazilian Archives of Digestive Surgery (ABCD) with several surgeons from Hospital das Clínicas and Brazil, which later became the official scientific agency of the Brazilian College of Digestive Surgery (CBCD). This spirit of national union concerning the specialty also enabled, in 1988, the idealization and foundation of the **Brazilian College of Digestive Surgery (CBCD)**, gathering specialists from all over Brazil. It was registered with the Brazilian Medical Association, the Brazilian Federal Council of Medicine, and competent bodies, with the specific determinations for obtaining the degree of specialist, in addition to the recognition of medical residency in the specialty by the National Medical Residency Commission (CNRM)^1^.

He was member of several national and international Medical Societies, notably the Brazilian Medical Association, the Brazilian Federation of Gastroenterology, the Brazilian College of Surgeons, the American College of Surgeons, the International Society of Diseases of the Esophagus, and the International Association for Gastric Cancer. In particular, his activities at the International Society for Diseases of the Esophagus (ISDE) are noteworthy, where he was president of the 2001 World Congress, held in São Paulo, Brazil.

He made numerous study trips inside and outside the Country. In Brazil, he participated in congresses, courses, and medical conferences in most states, visiting its main medical and university centers, where he had the opportunity to learn about the problems related to regional clinical pathology, medical assistance, professional issues, scientific research, and especially medical education.

He received numerous honors, including the title of **Doctor Honoris Causa** of the University of Goiás (Brazil), University of Coimbra (Portugal), and University of Milan (Italy).

He became Professor Emeritus at the Medical School of the University of São Paulo in 2000.

For those who knew and related to Prof. Pinotti, he was an authentic leader, who showed great dedication to undergraduate education, in particular by creating a center for educational training in surgery for fourth-year students, the so-called *grupo dos Zezinhos* (“group of *Zezinhos”*), who carried out their undergraduate research by instrumenting their surgeries and participating in clinical discussions. It is also worth noting his dedication to the 71 HCFMUSP Digestive Surgery residents trained under his guidance and to the 26 private Service residents at Hospital Nove de Julho. He actively participated in graduate studies advising 57 surgeons and several assistants, including the authors of this text.

We thank him on behalf of all of them, quoting his own words: “The mission for which we set ourselves has been achieved, the climax generates a state of tranquility as we have culminated in one of the fundamental actions of the University Professor of Surgery, which is to transmit what he knows.”
